# Herpes simplex virus and the lexicon of latency and reactivation: a call for defining terms and building an integrated collective framework

**DOI:** 10.12688/f1000research.8886.1

**Published:** 2016-08-19

**Authors:** Nancy M. Sawtell, Richard L. Thompson

**Affiliations:** 1 Division of Infectious Diseases, Cincinnati Children's Hospital Medical Center, Cincinnati, OH, USA; 2 Department of Molecular Genetics, Biochemistry and Microbiology, University of Cincinnati College of Medicine, Cincinnati, OH, USA

**Keywords:** herpes simplex virus, latency, reactivation

## Abstract

The field of herpes simplex virus (HSV) latency and reactivation has been marked by controversy, which is not unexpected considering the complexities of the biology involved. While controversy is an important tool for digging to the bottom of difficult issues, we propose that unproductive conflict in the field arises in part from poorly defined terminology and the need for a collective framework. The uses of advanced global molecular and next-generation sequencing approaches and an increasing array of *in vitro* model systems have provided new molecular-level insights into HSV latency and reactivation, with the promise of expanding our concepts of these processes. However, our current framework and language are inadequate to effectively integrate new data streams into the established theories. In this brief perspective, we look back into the past to examine when and how the lexicon of HSV latency and reactivation arose in the literature and its evolution. We propose to open a dialogue among investigators for the purpose of updating and clearly defining terms used to describe these processes and to build a collective integrated framework to move our field forward.

## Introduction

The intricately balanced relationship between humans and herpes simplex virus (HSV) type 1 has been honed over 6 million years of coevolution ^1^, the complex interactions of this lifelong partnership refined as hominids evolved into *Homo sapiens*. At its core, this relationship hinges on the ability of the virus to aggressively replicate in the epithelial cells at the site of infection, transport into the nervous system through the axons innervating the infection site, and enter a repressed state called latency. Periodically, the latent viral program in rare neurons is switched to the lytic cycle and infectious progeny are transported back to the body surface followed by rounds of replication in mucosal epithelium and virus shedding with the potential for transmission to new hosts. While significant progress in understanding these processes has been made, there remains much to discover and in this discovering, controversy has and will continue to arise.

**Figure 1.**
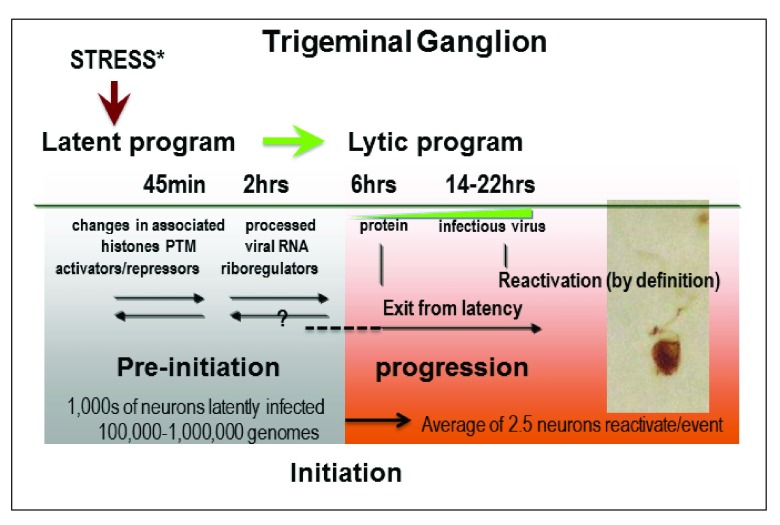
The process of herpes simplex virus (HSV) reactivation from latency. Some sensory neurons in the trigeminal ganglion (TG) of latently infected mice contain the viral genome, and the number of copies per neuron varies from 1 to more than 1000 ^57, 58^. A subset of these neurons express the latency-associated transcript locus that expresses a primary transcript processed into small and long non-coding RNAs as well as very low-level transcription from most or all of the viral genome ^52, 54, 59– 61^. Following stresses that can induce viral reactivation from latency, changes in the post-translational modification (PTM) of histone tails on the viral genome can be rapidly detected and processed viral RNAs (e.g. spliced) become more abundant within the first few hours, but viral proteins are not detectable (reviewed in 2– 5). Viral protein becomes detectable about six hours post-stress in very rare neurons, and we employ the term “initiation” to describe this event ^57^. By twenty-two hours post-stress, infectious virus can be detected in 50–70% of the TG. Photomicrograph shows rare neuron in the process of reactivating.

Partitioning the “process of reactivation” into a series of subevents may ultimately allow the development of improved models that can lead to testable hypotheses across the array of distinct model systems. This approach may prove particularly useful as we traverse the bridge between classic technology and the rapidly advancing ‘omic’ approaches. The following scenario of reactivation is drawn from the mouse ocular model and in this sense represents one facet but is used here for illustrative purposes. The application of advanced global molecular surveys has provided significant insights, including signature histone tail post-translational modifications associated with key regions of the latent viral genome and transcriptional activity from the viral genome during latency, including potential regulatory noncoding viral and host RNAs (reviewed in 2– 5). Changes detected by these global readouts occur rapidly post-reactivation stimulus, emphasizing that *many* latent viral genomes are linked to neuronal physiology and altered at this level from the pre-stimulus state. However, at the other end of this “reactivation process” is the production of infectious virus, which is generated at extremely low levels in the ganglion. The significance of this becomes apparent when we move from global to single cell analyses, as shown schematically in Figure 1. While infectious virus is measured as a global outcome, i.e. from the homogenized cell culture or tissue on permissive cell monolayers, the number of neurons expressing viral proteins (shown to correlate with infectious virus production ^6, 7^) is extremely rare. In the mouse trigeminal ganglion (TG) model following a reactivation stimulus *in vivo*, at the peak, 0.04% of latently infected neurons progress to viral protein expression (average of 2–4 neurons/TG) ^8, 9^ (note: while this number varies modestly with viral and mouse strain, the remarkable restriction is a common feature).

This outcome implies that events that negatively influence the process dominate and/or that downstream positive events occur rarely. Importantly, in between the derepression of the genome and associated increased transcriptional activity are likely to be multiple regulatory tiers. These regulatory tiers would function to transform a broad response (the “relaxing” of many latent viral genomes and associated “generalized” transcriptional activity) into the very rare neuron entering the lytic cycle and generating low levels of infectious virus. From this model, viewing the “reactivation process” as a complex event stream culminating in the rare reactivating neuron is useful. Further, we can consider the rare event “reactivation” as the intersection of a nested sequence of events. The possibility opens up that by comparing and contrasting increasingly refined “events” within this stream from diverse model systems, a picture of the components of the “reactivation process” will emerge.

Below we consider how language has evolved to describe the complex processes of latency and reactivation and how inadequate it seems to support our current understanding. There is controversy over the molecular events that “initiate” the “reactivation” of HSV from “latency”. Current hypotheses include the simultaneous induction of transcription of all viral genes of all kinetic classes ^10^, a wave of this simultaneous HSV transcription followed by transcription dependent on the potent virally encoded transactivator viral protein 16 (VP16) ^11^, the *de novo* expression of the immediate early ubiquitin E3 ligase infected cell protein 0 (ICP0), which derepresses or reactivates viral genomes ^12^, or alternately the *de novo* expression of VP16 followed by the familiar lytic cascade of VP16-induced viral gene transcription ^13^. The three words in quotes in the second sentence will be used to explore the idea that some of the current controversy in the literature is due to the way various groups employ the language of viral latency and reactivation to describe their results and draw their conclusions. Standardization of operational definitions of such terms might go far to eliminate confusion and controversy. This is not a new idea, and so a review of how the lexicon surrounding the interesting natural history of HSV disease developed and evolved through time is instructive (“history holds wisdom, despite the notion that the history of science bores most scientists stiff”, Sir Peter Medawar). Our goal is to begin a dialog with all scientists interested in these phenomena to develop a consensus of language to describe our collective findings across diverse model systems.

## Early definitions of viruses and viral latency

Studies in the 1930’s demonstrated that herpetic disease was caused by a “living agent” thought to be a filterable agent (e.g. not a bacterium). Grüter first showed transmission of human herpetic stromal keratitis to rabbit corneas ^14, 15^ and Loewenstein also demonstrated transmission to rabbits and subsequently transmission back to a human ^16, 17^ (reviewed in 18). In the 1950’s, the nature of filterable agents, also known as viruses, was still debated ^19^. Members of this class of pathogens were known to share common characteristics including the ability to replicate, cause disease, and in animals to engender immunity to subsequent infection. However, it was recognized that there were circumstances in which a host could be infected by a virus that replicated and yet show no signs of disease. Individual scientists used different terms to describe this, including “masked infection”, “occult infection”, “inapparent infection”, and “latent infection”. An attempt was made to clarify these terms at the Wisconsin meeting on Latency and Masking in Viral and Rickettsial Infections in 1957 ^20^. Six types of “latent” infection were described, including herpes virus infection in which “latency” was defined as the period of time in which skin was negative between outbreaks.

At this time, herpesvirus was considered to be a unique type of agent. It was noted that herpes lesions were restricted to individuals with pre-existing neutralizing antibodies and thus it was concluded that the affected individuals must have been previously infected. This was the only known example where a virus persisted in definable cells (thought then to be skin) without indication of its existence between occasional outbreaks of clinical activity. Interestingly, this coincided with the time that the concept of lysogeny in bacteria was occupying the minds of microbiologists and much focus centered on the fact that lysogenized bacteria were refractory to subsequent superinfection. Indeed, it was thought that HSV infection in humans might be similar to lysogeny of bacteria ^21, 22^. Clearly HSV was not a classic lysogen because during "recrudescence", many thousands of presumably herpes "lysogenized" cells became infected, forming the visible lesion of "recurrent disease" and thus these infected cells must not be refractory to superinfection.

## Animal models of HSV and the evolution of the terms “latency” and “reactivation” (1918–1972)

The natural history of herpesvirus infection was well recognized at this time as a primary aphthous stomatitis occurring sometime before puberty followed by repeated attacks of "fever blisters" precipitated by a variety of known and unknown stimuli that “reinitiates” infection. The terms "recrudescence" and "recurrent disease" were employed to define these lesions in humans from which infectious virus could be isolated. The understanding of this inexplicable infection was hampered, as asserted by Burnet in 1960 in that “No laboratory model of herpes in man has yet been described” ^22^. Although the concept that latent virus in the skin was the source of recurrent lesions was prevalent at this time, elegant early studies by Goodpasture, largely ignored, had suggested twenty years earlier a link between sensory ganglionic neurons and recurrent herpes infection ^23^. Good and Campbell employed Perdrau’s model of herpetic encephalitis in pre-immunized rabbits ^24, 25^ to demonstrate the “precipitation” of herpesvirus encephalitis in rabbits following anaphylactic shock ^26^. Schmidt and Rasmussen sought to follow up on their work by exploring alternate methods to “precipitate” herpes ^27^. They employed the pyrogen pyromen to induce moderate to high fever for 36 hours as well as cortisone, acetate, and glutathione to no effect. However, encephalomyelitis was “precipitated” in 60% of the rabbits given intramuscular injections of adrenalin. Herpesvirus was detected in all six of these rabbit brains.

Early herpes virologists stored their stocks of virus as bits of infected rabbit brain tissue in glycerin solutions in an icebox. Perdrau noted that exclusion of air greatly increased the length of time such solutions maintained their “activity” as defined by the capacity to cause disease in rabbits. Oxidation destroyed herpesvirus “activity”, but the herpes stock could be “reactivated” by subsequent reduction ^28^. Rasmussen speculated “that temporary vasoconstriction resulting from increased adrenalin output, could produce a local anoxia and consequent “reactivation” of residual herpes virus” ^27^. Thus, Rasmussen changed “precipitation” to “reactivation”. This was quite an astounding change from a term implying some form of regeneration of infectious virus to one that implies (re)-activation of a pre-existing viral agent. The following year, Kilbourne and colleagues showed that an Arthus type reaction to horse serum could induce recurrent corneal disease in rabbits from which active virus could be obtained ^29^. While the authors gave credit to Good and Campbell for the first demonstrations of what they now called “reactivation”, this ocular model fulfilled most of the criteria for a true laboratory model of herpes infection in humans. Note that the term “reactivation” was already in use in the clinical literature, but this is the earliest herpesvirus basic science manuscript we have found with “reactivation” in the title. At this time (1961), “reactivation” meant a recurrent lesion from which viable herpesvirus could be isolated. A great deal of indirect evidence was mounting to support that the sensory ganglion was the site of herpesvirus latency. About a decade later, Stevens and Cook demonstrated that latent HSV-1 could be “reactivated” out of “quiescently” infected mouse dorsal root ganglia, confirming this possibility ^30^. Thus, in 1971, Stevens modified the term “reactivation” to include virus recovered from axotomized sensory ganglia explanted into culture. His most important control was to show that infectious virus or complete virions (by electron microscopic examination) were not already present in the ganglia but “reactivated” upon explant. Stevens reasserted the idea that reactivation from latency was the *de novo* production of infectious virus in a tissue in which pre-existing virus did not exist. This use of “reactivation” was considered to be controversial by some.

## Tissue culture models of latency

During the 1950s, there was a significant interest and study of various latent infections in tissue cultures. Some thought these to be important models of latent human infections and the possible origin of human cancer, while others regarded the phenomenon as a laboratory artifact (reviewed by Ginsberg in 31). Herpes virologists of the time weighed in heavily on the latter idea. This debate still continues today to some extent, but a more scientifically sound approach is to recognize that all models have intrinsic differences. Consideration of the advantages and disadvantages of each model allows findings to be placed in the proper context and thus integrated with outcomes from other models. In 1972, O’Neill and colleagues infected human cells with HSV-2 in the presence of cytosine arabinoside (ara-c) and established cultures that could be maintained without apparent virus-induced cytopathic effect (CPE) for five days. Upon removal of the drug, CPE was detected after a lag of five to six days and they defined this period of time as “latent” and the re-emergence of replicating virus as “reactivation” ^32^. Many leaders in the field of HSV latency objected quite strongly to this redefinition of these terms. While one may argue about terminology, there can be no doubt that such cultures have provided an abundance of information on the biochemical roles played by key viral proteins and how they might function during the processes of latency and reactivation.

The types of cell cultures employed to model latency have also evolved. In 1987, Wilcox promoted the use of primary neuronal cultures in the presence of nerve growth factor (NGF) as a culture-based model of latency and demonstrated that removal of NGF resulted in “reactivation” of virus in these cultures ^33^. Resistance to the use of these terms in cultured cells ran high, even after Wilcox and colleagues demonstrated certain latent-like characteristics such as the expression of the latency-associated transcripts in some neurons ^34^. At the end of the 80’s, Leib, Schaffer, and colleagues employed a hybrid system in which the mouse corneal model was used and TG from mice latently infected with diverse mutant or wild-type viruses were removed, dissociated, and dispersed in culture plates. They extended the term “reactivation” to the recovery of virus following superinfection with wild-type or mutant viral isolates. One conclusion of this study was that the immediate early gene protein ICP0 was required for efficient “reactivation” ^35^. In a parallel set of experiments, Cai and Schaffer employed DNA from ICP0 mutants in transfection studies and found that “ICP0 plays a critical role in the de novo synthesis of infectious virus following transfection” ^36^. These and certain other manuscripts from this group are often cited by others as demonstrating that ICP0 plays an important role in the “initiation” of reactivation. It should be noted that Schaffer and co-workers did not claim this, consistent with the meticulous use of language in these manuscripts.

## VP16 and ICP0 and their roles during acute infection of cultured cells and reactivation from latency

HSV lytic viral replication is “initiated” in an unusual way. This virus carries its own transcription factor within the virion and this protein cooperates with host cell proteins to initiate the transcription of the five viral immediate early genes. There is nearly universal acceptance of the term “initiation” of lytic infection for this process. In the early 1980’s, Preston and colleagues identified the viral gene that encodes the protein that “initiates” HSV lytic infection ^37^. They later made viral mutants in which this transactivation function was disrupted. One of these mutants, in1814, was replication competent in tissue cultures infected at a high multiplicity of infection (MOI) but very defective for inducing virus plaques at low MOI ^38^. This protein, termed VP16, was thought to be an obvious possible mechanism for the “initiation” of reactivation. With alacrity, Fraser and colleagues tested Preston’s mutant in the mouse model of latency using the axotomy/explant reactivation model of Stevens ^39^. Surprisingly, the mutant reactivated in explant quite well. Roizman’s group attempted to express VP16 in TG neurons in transgenic mice or with an inducible promoter in the virus and concluded that VP16 expression did not perturb the establishment of latency or induce reactivation from latency ^40^. These results led the field to abandon the idea that VP16 initiates reactivation and to refocus on other potential mechanisms including the work discussed above on the immediate early gene protein ICP0. We now know that *de novo* expression of VP16 (expression of VP16 in the absence of other viral proteins) is a requisite, precipitating event that can initiate reactivation in TG neurons *in vivo* following stress ^13, 41^. VP16 is not required for “reactivation” following axotomy and explant of ganglia into culture, one difference between these models ^8^. It is not clear why the experiments of Sears *et al*. failed to demonstrate this activity, but it may be that the VP16 protein was not expressed in the appropriate neurons.

Understanding how HSV reactivation from latency is initiated is an important goal, but it is clear that there is not a general agreement on what “initiation of reactivation” means. One fading controversy we address here for illustrative purposes is about the roles that VP16 and ICP0 play in the process of the “initiation” of HSV reactivation from latency. The hypothesis that ICP0 might “initiate” reactivation from latency comes largely from the work performed in “quiescently” or “ *in vitro* latently” infected cultures. Many groups have made valuable contributions and it is not possible to cite them all here, but this important work has been recently reviewed ^42, 43^. Utilizing quiescently infected fibroblast cultures, which Preston termed “ *in vitro* latency”, it was concluded in 1997 that the latent genomes failed to respond to VP16 but did respond to ICP0 ^44^. However, ten years later, Preston revisited this approach and demonstrated that ICP0 was not required in this system ^45^, thereby showing the quiescent genomes were actually responding to VP16.

What is clear is that the ectopic expression of ICP0 can modify the chromatin marks present on quiescent/latent viral genomes in tissue culture systems and can derepress these genomes such that the silenced promoters are expressed ^46– 49^. It turns out that ICP0 is not a “promiscuous transactivator” but rather a E3 ubiquitin ligase with many targets that mediate its functions through its interactions with these targets ^50^. These activities lend credence to the idea that the *de novo* expression of ICP0 might initiate viral reactivation from latency ^51^. However, *de novo* expression of ICP0 has not yet been demonstrated, and viral mutants in which ICP0 is deleted do exit the latent state and initiate reactivation (as defined by the production of viral proteins) *in vivo* following hyperthermic stress ^52^. Importantly, in the absence of ICP0, neurons in which latency is exited *in vivo* do not progress to produce infectious virus ^52^. As concluded by Everett in 2011, “It is likely that the initial events of reactivation leading to viral gene expression are not ICP0 dependent, but that ICP0 is required to allow progression from this early stage of reactivation to a productive infection …” ^53^. There is no argument that these two “nonessential” viral protein functions (VP16 transactivation and ICP0 E3 ubiquitin ligase) are both extremely important very early during reactivation from latency; indeed, they are seemingly absolutely essential *in vivo*. The only argument is whether one or the other initiates HSV reactivation, and this depends in part on how one defines the term “initiate” reactivation.

An obvious candidate for the earliest events during reactivation is transcription of viral lytic phase genes. However, during latency, the transcription of most, if not all, of the HSV-1 genome occurs at low levels ^52, 54^. Attempts to examine the earliest events in reactivation using sensitive methods like semi-quantitative reverse transcription polymerase chain reaction were not successful because subtle changes at early times post-induction of reactivation were hard to parse out from this background. More recent findings show that a wave of transcription from many or all regions of the genome occurs under conditions that result in viral reactivation in several systems ^10, 11^. This transcription is likely the result of the “relaxing” of the chromatin structure on the latent viral genomes in response to stressors ^2– 5^. These changes must be occurring in the majority of latent genomes to be observable, but they are reversible and not sufficient of themselves to cause progression to virus production. We propose that a term like “pre-initiation” could encompass such reversible changes ( Figure 1). We chose to use the term “initiation” for the expression of detectable viral protein in neurons in latently infected ganglia, either spontaneously or following a stressor that induces viral reactivation. Detectable viral protein production (initiation) occurs in only one or very few neurons in a TG *in vivo* following hyperthermic stress ^55, 56^. It seems likely that there is a threshold of viral protein production that leads to progression through the lytic cycle. The relationship between detectable viral protein expression and “progression” (see Figure 1) to virus production remains unknown, but they are highly correlated ^6, 56^. Progression to the production of infectious virus is dependent on most viral genes, and it is likely that all viral genes (and non-coding RNAs) play important roles in this process. We do not yet know the point of no return during progression to virus production, and it may be that some initiation events are abortive. Of special interest will be the discovery and characterization of novel viral or host functions that specifically modulate the process of viral reactivation because these may serve as therapeutic targets.

As common terms are developed and defined in diverse models, it will be important to consider context. Common models include intact human hosts, perturbed human host (i.e. diseased, damaged, surgical patients, dead hosts), intact animal host (and those similarly perturbed such as axotomized and explanted latently infected sensory ganglia, various invasive and non-invasive triggers that induce viral reactivation from latency), and human and animal cell cultures in their diversity (transformed and highly mutated like HeLa cells, primary or secondary cell lines, primary neurons, neuronal-like cells that are or are not “differentiated”, etc.). It is our hope that collectively we can begin to define a lexicon of latency and an integrated framework that allows us to argue about the science, and not the language or the model. Toward this goal, we propose a forum where interested scientists and students can discuss such issues. Join us on the moderated Facebook page at “virtual herpesvirus workshops”.

## Abbreviations

CPE, cytopathic effect; HSV, herpes simplex virus; ICP0, infected cell protein 0; MOI, multiplicity of infection; NGF, nerve growth factor; TG, trigeminal ganglion; VP16, viral protein 16.
